# Excited‐State Dynamics in All‐Polymer Blends with Polymerized Small‐Molecule Acceptors

**DOI:** 10.1002/advs.202301931

**Published:** 2023-06-04

**Authors:** Ziran Liu, Qian Li, Lulu Fu, Jide Wang, Jing Ma, Chunfeng Zhang, Rui Wang

**Affiliations:** ^1^ Key Laboratory of Oil and Gas Fine Chemicals Ministry of Education & Xinjiang Uygur Autonomous Region School of Chemical Engineering and Technology Xinjiang University Urumqi 830046 China; ^2^ National Laboratory of Solid State Microstructures School of Physics, and Collaborative Innovation Center for Advanced Microstructures Nanjing University Nanjing 210093 China; ^3^ School of Materials Science and Engineering Qilu University of Technology (Shandong Academy of Sciences) Jinan 250000 China; ^4^ Institute of Theoretical and Computational Chemistry Key Laboratory of Mesoscopic Chemistry of MOE School of Chemistry and Chemical Engineering Nanjing University Nanjing 210093 China; ^5^ Institute of Materials Engineering Nanjing University Nantong Jiangsu 226019 China; ^6^ College of Physics Nanjing University of Aeronautics and Astronautics, and Key Laboratory of Aerospace Information Materials and Physics (NUAA) MIIT Nanjing 211106 China

**Keywords:** charge generation and recombination, excited‐state dynamics, polymerized small molecular acceptor, transient absorption, Ï€‐Ï€ stacking

## Abstract

Polymerizing small‐molecular acceptors (SMAs) is a promising route to construct high performance polymer acceptors of all‐polymer solar cells (all‐PSCs). After SMA polymerization, the microstructure of molecular packing is largely modified, which is essential in regulating the excited‐state dynamics during the photon‐to‐current conversion. Nevertheless, the relationship between the molecular packing and excited‐state dynamics in polymerized SMAs (PSMAs) remains poorly understood. Herein, the excited‐state dynamics and molecular packing are investigated in the corresponding PSMA and SMA utilizing a combination of experimental and theoretical methods. This study finds that the charge separation from intra‐moiety delocalized states (i‐DEs) is much faster in blends with PSMAs, but the loosed *π*–*π* molecular packing suppresses the excitation conversion from the local excitation (LE) to the i‐DE, leading to additional radiative losses from LEs. Moreover, the increased aggregations of PSMA in the blends decrease donor: acceptor interfaces, which reduces triplet losses from the bimolecular charge recombination. These findings suggest that excited‐state dynamics may be manipulated by the molecular packing in blends with PSMAs to further optimize the performance of all‐PSCs.

## Graphical abstract

In blends with polymerized small‐molecule acceptors (PSMAs), the *π*–*π* molecular stacking is reduced, which decreases the conversion from local excitations to intra‐moiety delocalized excitations in PSMAs and leads to radiative loss from local excitations during charge generation. Moreover, triplet losses of charge recombination are suppressed because of the reduced donor: acceptor interfaces in PSMA based blends.

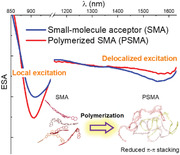

## 1 Introduction

All‐polymer solar cells (all‐PSCs) have attracted tremendous interest mainly because of their mechanical flexibility, morphology stability, and low cost.^[^ ^1^, ^2^, ^3^, ^4^, ^5^, ^6^, ^7^ ^]^ Owning to the strategy of polymerizing small molecular acceptors (SMAs) to construct new polymer acceptors,^[^ ^8^, ^9^ ^]^ the power conversion efficiency (PCE) of all‐PSCs with polymerized SMAs (PSMAs) have increased dramatically to above 18%,^[^ ^10^, ^11^, ^12^, ^13^, ^14^, ^15^ ^]^ recently. The PCE improvement of all‐PSCs with PSMAs are often explained by preserving the advantage (including strong near infrared absorption, narrow bandgap, and adjustable electronic levels) of SMAs as well as the merits (such as superior long‐term stability, better electron mobility, and good film‐forming property) of the polymers.^[^ ^4^, ^9^, ^16^ ^]^ Nevertheless, the PCEs of all‐PSCs still lag behind their polymer: SMA counterparts.^[^ ^17^, ^18^, ^19^, ^20^, ^21^ ^]^ To further improve the device performance, the excited‐state dynamics, which sets the intrinsic upper limit of photon‐to‐current conversion,^[^ ^22^, ^23^ ^]^ needs to be explored in PSMA‐based organic photovoltaic (OPV) blends.

In general, the properties of pivotal excited state dynamics in OPV materials are determined by the molecular structure and packing.^[^ ^24^, ^25^, ^26^ ^]^ After polymerization, while the molecular structures reserve in PSMAs, the molecular packing can be largely modified by the linkages with covalent bonds. Therefore, the characters of localized and intra‐moiety delocalized excitations in blends with PSMA may differ from that of their SMA counterparts.^[^ ^27^, ^28^ ^]^ In state‐of‐the‐art SMA based OPV blends, intermolecular *π*–*π* stacking have been argued to regulate the vital charge generation and recombination dynamics.^[^ ^29^, ^30^ ^]^ Uncovering the relation between these microstructures and excited‐state dynamics is also essential to fully understand the power conversion mechanism in PSMA based OPV blends, which, however, is still elusive.

In this work, we systematically study the excited‐state dynamics together with the microstructure of molecular packing in OPV blends with corresponding PSMAs and SMAs using broadband transient absorption spectroscopy, grazing incidence wide‐angle X‐ray scattering (GIWAXS), and Molecular‐dynamics simulations. We find that the loosened *π*–*π* stacking polymer chains in PSMA can suppress the conversion from local excitations (LEs) to weakly bound intra‐moiety delocalized excitations (i‐DEs), which leads to more radiative loss from LE during the i‐DE mediated charge separation in OPV blends with PSMA. Moreover, the non‐radiative charge recombination of triplet loss is found reduced in PSMA‐based blends due to the decreased donor: acceptor (D: A) interfaces. The results suggest that manipulating the *π*–*π* stacking of the PSMAs in blends can optimize the excited‐state dynamics of LE to i‐DE conversion, which could potentially improve the performance of all‐PSCs.

## 2 Results and Discussion

**Figure 1:**
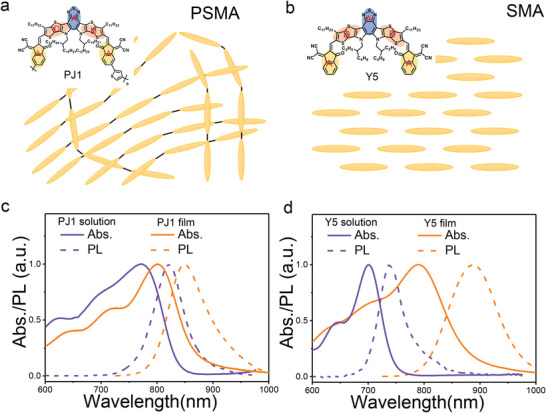
a) Schematic diagram of the molecular structures and packing of the corresponding PSMA and b) SMA aggregations. The related absorption and photoluminescence spectra of typical PSMA PJ1 and SMA Y5 in solutions and films are compared in (c,d).

To study the effect of SMA polymerization, we choose a model system consisting of a PSMA PJ1 and a SMA Y5 (**Figure** **1**a,b).^[^ ^31^ ^]^ Considering the structures of containing the same molecular fragments of A‐D‐A’‐D‐A, Y5 can be regarded as the building block of PJ1. In PJ1 chains, the SMA subunits are head‐to‐tail linked by thiophenes (Figure 1a),^[^ ^32^ ^]^ which is different from the staggered packing of molecules in Y5 films (Figure 1b).^[^ ^33^ ^]^ The different micromorphologies of molecular arrangement can change the *π*–*π* interaction in PSMAs and SMAs. To acquire the molecular packing effect on the excited‐ state properties, we compare the absorption and emission spectra of PJ1 and Y5 solutions and neat films in Figure 1c,d. In the solution samples, the interchain interaction of PJ1 and intermolecular interaction of Y5 induced by molecular packing are largely reduced. Thus, the absorption spectra of PJ1 and Y5 solutions are both blueshifted in compression with that of the neat films. Moreover, the spectral blueshift of PJ1 is less significant than Y5, suggesting the interchain *π*–*π* interaction of PJ1 is weaker than that of Y5 in neat films. In addition, the shift between the absorption and emission spectra are more pronounced in the films as compared to that in the solutions (Table S1, Supporting Information), which indicates larger nuclear displacements of the excited states in PJ1 and Y5 films.^[^ ^28^, ^34^, ^35^ ^]^ Furthermore, the redshift of emission peak for Y5 film is much more significant than that for PJ1 film (Table S1, Supporting Information). In OPV materials, the excited states may be hybridized by both LE and charge‐transfer (CT) excitations.^[^ ^36^ ^]^ Considering the CT state generally exhibits a large displacement of the potential energy surface between the CT state and the ground state, the larger peak displacement between the absorption and emission spectra suggests the proportion of the CT excitation is higher in the Y5 film than that in the PJ1 film. These results suggest the relatively loose microstructure of polymer chains in the PJ1 film may decrease the strength of *π*–*π* interaction and reduce the CT character of excited states as compared with Y5 film.

**Figure 2:**
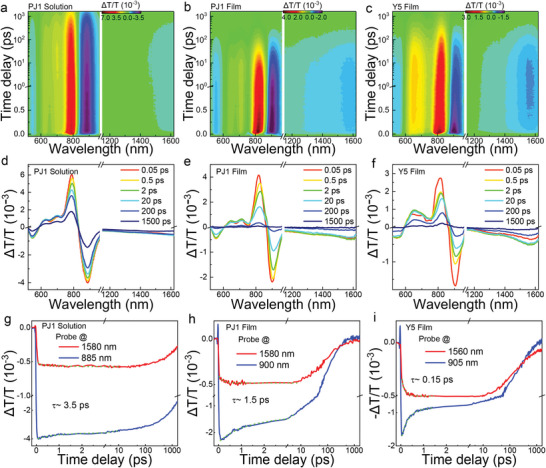
Transient absorption data recorded from samples of a) PJ1 solution, b) PJ1 film, and c) Y5 film. Transient absorption spectra at different time delay of d) PJ1 solution, e) PJ1 film, and f) Y5 film, respectively. The dynamics of LE ≈900 nm probe and i‐DE ≈1580 nm probe of the three samples are compared in (g–i), respectively.

The excited‐state evolution is also affected by different molecular packing in the PSMA and SMA, which is studied by the broadband transient absorption (TA) spectroscopy (details in the Supporting Information). The experimental results of PJ1 and Y5 neat films and solution samples are compared in **Figure** **2** and Figure S1 (Supporting Information). The bleach signals ≈800 nm can be assigned to the ground state bleaching (GSB) according to the absorption spectra. A distinct excited state absorption (ESA) signal ≈900 nm is recognized in all the four samples including Y5 solution of a single molecule (Figure 2a–c; Figure S1, Supporting Information), which can be safely ascribe to the Frenkel‐type LEs. In the near infrared range, another ESA signal appears ≈1500–1600 nm in the samples of PJ1 solution, PJ1 film and Y5 film but disappears in Y5 solution. In contrast to the Y5 solution where the intermolecular interaction can be ignored, a fraction of interaction between SMA subunits can still be preserved because of the intertwined polymer chains in PJ1 solution.^[^ ^37^, ^38^ ^]^ The different occurrence of the near infrared ESA signals in the solution and film samples suggests the excitation is related to the interaction between SMA molecules or subunits, which can be assigned to the i‐DE as reported previously in a similar SMA of Y6.^[^ ^28^ ^]^ The interaction induced hybridization of these states leads to the formation of a portion of i‐DE within the pulse duration, while more i‐DE can be generated by conversion from LE in the following picoseconds (Figure 2d–f). Nevertheless, the generation dynamics and fraction of the i‐DE alter with different molecular packing conditions. We compare the corresponding decay and generation dynamics of LEs probed at ≈900 nm and i‐DEs probed at ≈1580 in Figure 2g–i. In the PJ1 film, the lifetime of LE to i‐DE conversion is characterized as ≈1.5 ps (Figure 2h), which is slower than that (≈0.15 ps) of the Y5 film (Figure 2i). In addition, the amplitude ratio of i‐DE signal is also decreased in the PJ1 film, suggesting less i‐DE is formed. The formation of i‐DE is further suppressed in the PJ1 solution, with the conversion lifetime of ≈3.5 ps (Figure 2g). In PJ1 solution, the backbones of PJ1 remain unchanged but the packing between the subunits of polymer chains is largely released, which vanishes the effect of molecular structures and side chains on the exited‐state dynamics.^[^ ^39^, ^40^ ^]^ Therefore, the decreased LE to i‐DE conversion in PJ1 solution suggests the suppressed *π*–*π* molecular packing can prevent the excitation transformation in PSMAs. To further confirm this, we perform the TA measurements on the PJ1 solution with different temperatures. By increasing the temperature, the *π*–*π* packing between the entangled polymer chains of PJ1 in the solution will be further reduced, which is consistent with gradually blue‐shifted absorption spectra of PJ1 solution with higher temperatures (Figure S2, Supporting Information). Moreover, we find the recorded TA signals of i‐DE are also suppressed with higher temperatures (Figure S2, Supporting Information). These results strongly demonstrate the reduced *π*–*π* stacking in PSMA strongly suppresses the formation from LE to i‐DE in the acceptors, which may further affect the following charge generation and recombination dynamics in OPV blends.

**Figure 3:**
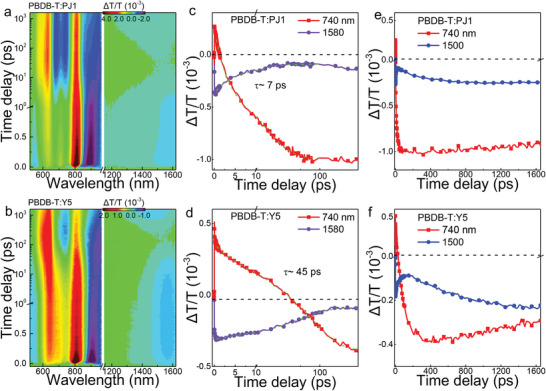
a,b) TA data recorded from blended films of PBDB‐T: PJ1and PBDB‐T: Y5. The extracted temporal dynamics of i‐DE ≈1580 nm probe and CS ≈740 nm probe of PBDB‐T: PJ1and PBDB‐T: Y5 are shown in (c,d), respectively. The extracted charge generation lifetime is much shortened in PBDB‐T: PJ1 (≈7 ps) in comparison with that of PBDB‐T: Y5 (≈45 ps). Kinetic curves probed at the characteristic wavelengths showing the charge recombination and triplet formation dynamics of e) PBDB‐T: PJ1and f) PBDB‐T: Y5, respectively.

We further study the impact of SMA polymerization on the dynamics of charge generation and recombination in OPV blends. We select a proper polymer donor of PBDB‐T (Figure S3, Supporting Information) to blend with the two acceptors of PJ1 and Y5, respectively. Upon excited at 800 nm, the excitations in PJ1 and Y5 are selectively populated that can contribute to free charges via the hole transfer channel. To monitor the charge dynamics after hole transfer, we compare the TA spectra recorded from the PBDB‐T: PJ1 and PBDB‐T: Y5 blended films in **Figure** **3**a,b. In both the two blends, we observe i‐DE mediated charge separation processes. Upon photon excitation, the ESA signals of i‐DE ≈1580 nm in the two blends are both recognized, which is consistent with the transient spectra of neat PJ1 and Y5 films (Figure 2). The following decays of the i‐DEs are much faster than that of neat films, demonstrating i‐DEs are involved in the hole transfer process (Figure S4, Supporting Information). Simultaneously, we find a gradually increase of new ESA bands ≈740 nm with the decay of i‐DE, suggesting a new species has been generated via hole transfer. In addition, the generated new species are long‐lived together with both the GSB signals of the donor and acceptor (Figure 3a,b), which can be assigned to the charge separated (CS) state containing free electrons and holes.^[^ ^28^, ^41^ ^]^ Therefore, the charge generation dynamics of PBDB‐T: PJ1 and PBDB‐T: Y5 can be represented by the corresponding decay and rise of i‐DE and CS states probed at 1580 and 740 nm, respectively (Figure 3c,d). By exponential fittings, the extracted charge generation lifetime of PBDB‐T: PJ1 is ≈7 ps, which is much shorter than that (≈45 ps) of PBDB‐T: Y5. The faster charge separation in PBDB‐T: PJ1 suggests that, in PJ1, the i‐DE state may have higher energy with delocalization along the conjugated polymer chain, which may decrease the binding energy and facilitate the charge generation in blends with PSMAs. Nonetheless, we find dynamics of LE is nearly unchanged in the first 10 ps (Figure S5, Supporting Information), indicating LE cannot directly involve in the hole transfer. In PJ1, the slower conversion from LE to i‐DE may lead to addition radiative loss from LE, which is confirmed by the reduced fluorescence quenching in PBDB‐T: PJ1 blend (Figure S6, Supporting Information).

In OPV blends with nonfullerene acceptors, bimolecular recombination of photo‐induced charges is an important loss channel.^[^ ^42^, ^43^ ^]^ Such a nonradiative recombination is significantly reduced after SMA polymerization. The bimolecular recombination of free charges can form charge transfer states with spin singlet or triplet (^1^CT, ^3^CT) at D: A interfaces.^[^ ^44^, ^45^ ^] 3^CT can continuum relax to the low‐lying triplet state in the donor or acceptor, reducing the device open‐circuit voltage.^[^ ^46^ ^]^ In both PBDB‐T:PJ1 and PBDB‐T:Y5 blends, we identify the non‐radiative charge recombination of triplet formation processes. At long time scale (200–1500 ps), the ESA signals of CS states probed at 740 nm start to decay with the building up of new ESA features probed at 1500 nm in the two blends (Figure 3a,b), which indicates a new excited‐state species is formed during the recombination of free charges. The newly‐generated ESA band ≈1500 nm are from triplet states in the acceptors as characterized by triplet sensitization experiments on PJ1 and Y5 (Figure S7, Supporting Information).^[^ ^43^, ^47^ ^]^ Although the triplet generation loss channels are both observed in the two blends with PSMA and SMA, the triplet generation dynamics show remarkable difference (Figure 3e,f). The relative ESA feature of triplets at ≈1500 nm of PBDB‐T: PJ1 blend is markedly reduced in the TA spectrum at long time delay of 1000 ps (Figure 3e,f). The kinetics of charge recombination is also reduced at 740 nm probe (Figure 3e,f). The picosecond triplet generation can directly affect free charge recombination dynamics at nanosecond timescale. We compare the nanosecond‐resolved TA results recorded from PBDB‐T: PJ1 and PBDB‐T: Y5 in Figure S8 (Supporting Information). The free charge lifetime is much prolonged in the blend with PJ1, which is consistent with the suppressed triplet loss channel. The above results suggest that SMA polymerization can suppress the non‐radiative charge recombination of triplet formation, extend the free charge lifetime, and potentially reduce the voltage loss.

**Figure 4:**
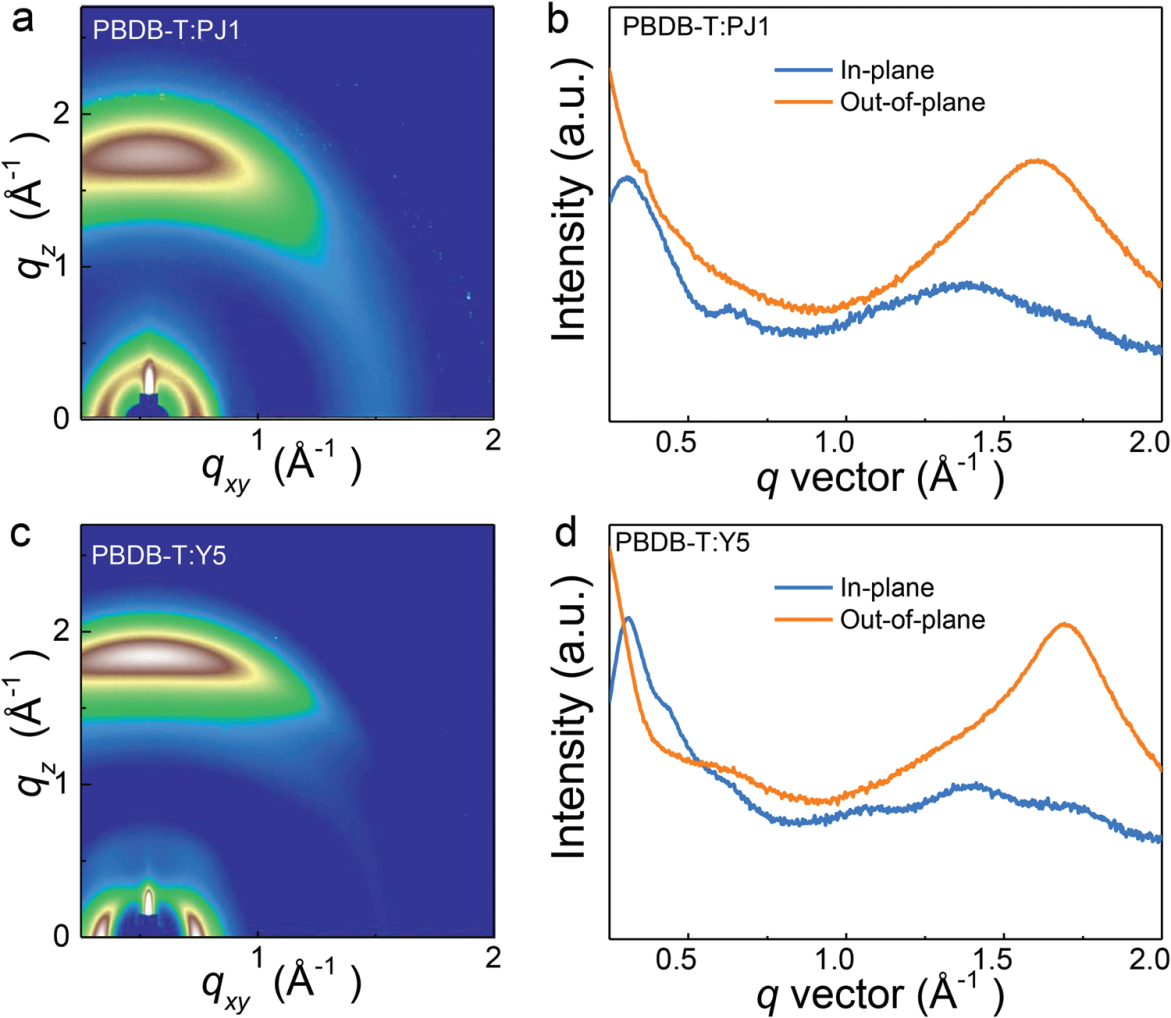
a,c) GIWAXS results and b,d) corresponding in‐plane and out‐of‐plane line‐cuts of PBDB‐T:PJ1and PBDB‐T:Y5 blends.

While we have observed the significant SMA polymerization effect on excited‐state dynamics, it is important to reveal the connection between the excited‐state dynamics and the molecular packing structures in PSMA and SMA.^[^ ^48^, ^49^ ^]^ The molecular packing information of blend films of PSMA and SMA checked by GIWAXS is performed in **Figure** **4**. In both blends with PJ1 and Y5, we observe the signatures of lamellar stacking and *π*–*π* stacking. The lamellar stacking diffractions are obvious in the in‐plane directions with *q* ≈ 0.28 Å^−1^ (lamellar stacking distance, *d*_l_ ≈ 22 Å). In the out‐of‐plane direction, clear peaks of 010 diffraction are found ≈1.60 Å^−1^, corresponding to a *π*–*π* stacking distance of ≈3.93 Å. The acquired *π*–*π* stacking and lamellar stacking parameters are consistent with that of neat PJ1 and Y5 films (Figure S9, Supporting Information), confirming the signals mainly come from the acceptors. Although the molecular packing distance is similar of PJ1 and Y5, the crystal coherent lengths (CCL) show remarkable differences (Table S2, Supporting Information). The extracted CCL of PBDB‐T: PJ1 is 40.8 Å for lamellar stacking and 19.3 Å for *π*–*π* stacking, which is significantly shorter than that of PBDB‐T: Y5 of 88.6 and 29.6 Å, respectively. As electronic coupling is mainly determined by the short *π*–*π* stacking,^[^ ^30^ ^]^ the superior crystallization of SMA may stabilize the delocalized excitations of i‐DE, which is consistent with the faster and larger fraction of i‐DE generation in SMA films.

**Figure 5:**
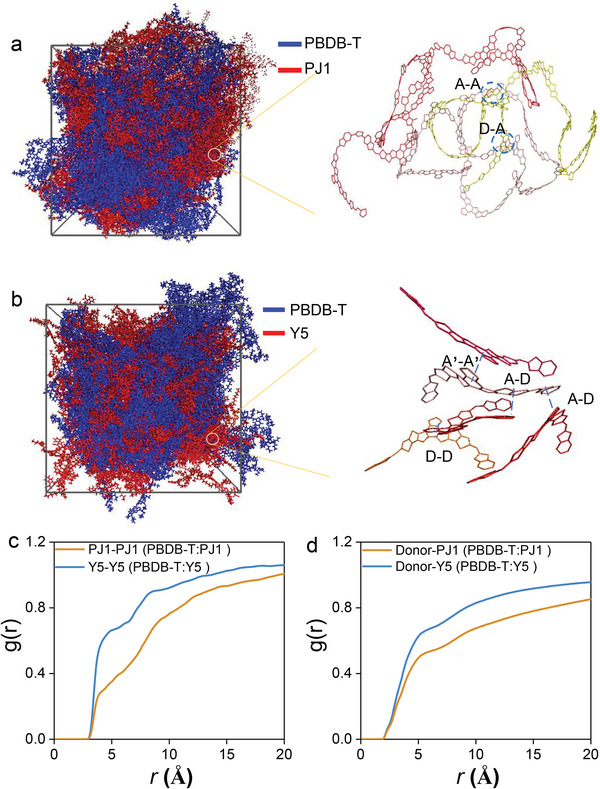
MD simulations of a) PBDB‐T: PJ1 and b) PBDB‐T: Y5 based systems, where the blue and red colors correspond to donor and acceptor, respectively. The enlarged picture shows the stacking between neighboring acceptor chains or molecules. Radial distribution function g(r) for the c) center‐of‐mass of acceptor subunits (molecules) and d) center‐of‐mass of the donor or acceptor subunits (molecules) in simulated PBDB‐T: PJ1 and PBDB‐T: Y5 blends. The RDFs of the PBDB‐T: Y5 film are higher than those of PBDB‐T: PJ1 blend, confirming that the *π*–*π* stacking and D: A interface are both suppressed in the blend with PSMA.

We further perform Molecular‐dynamics (MD) simulations to verify the influence of SMA polymerization on the microstructures of molecular packing of the two blends based on PJ1 and Y5 (**Figure** **5**). The polymer chains of PJ1 can entangle with each other in the blend, exhibiting higher phase separation in comparison to Y5 (Figure 5a,b).^[^ ^50^ ^]^ To exhibit the molecular packing situations, we calculate the radial distribution functions (RDFs), g(r), for both PJ1 and Y5 in the blends.^[^ ^51^ ^]^ The RDF data of all the molecular fragments (A, D and A’) in PJ1 and Y5 are compared in Figure S10 (Supporting Information). We find the g(r) functions of all the six packing conditions show a sharp increase ≈3.8 Å, which is consistent with the *π*–*π* stacking distance acquired by GIWAXS. To gain more insights of different *π*–*π* packing in the blends with PJ1 or Y5, we compare the g(r) functions by taking the center‐of‐mass of every subunit (molecule) of PJ1 (Y5) as the reference particles (labeled as PJ1‐PJ1 and Y5‐Y5) in Figure 5c. Considering the *π*–*π* packing and lamellar stacking distances of PJ1 or Y5 are ≈4 and 22 Å (Table S2, Supporting Information), the lower values of g(r) when r < 10 Å suggests a weaker *π*–*π* packing of PJ1 (Figure 5c). We also calculate the RDFs between the donor and acceptors to investigate the interface in the blends with PJ1 and Y5 by taking the center‐of‐mass of the subunit of PBDB‐T as one particle and the center‐of‐mass of the subunit (molecule) of PJ1 (Y5) as the reference particle (labeled as Donor‐PJ1 and Donor‐Y5 in Figure 5d). The lower g(r) of Donor‐PJ1 in the blend indicates the interfaces are largely reduced in PBDB‐T: PJ1, which may arise from the stronger aggregation of polymer chains. Moreover, the loosed *π*–*π* packing and decreased D: A interfaces in the blend with PJ1 are also confirmed by smaller integration of g(r) over the distribution for PJ1‐PJ1 (Y5‐Y5) and Donor‐PJ1(Y5) (Figure S11, Supporting Information). The MD simulation results clearly show that the *π*–*π* stacking and D: A interface are both suppressed in the blend with PSMA, which may strongly relate to the excited state dynamics. The weaker *π*–*π* interactions in PJ1 may reduce the excitation delocalization and thus result in the slower LE to i‐DE conversion. Meanwhile, the reduced D: A interfaces can suppress the encounter of free charges, which explains the decreased triplet formation loss in PBDB‐T:PJ1.^[^ ^52^ ^]^

The uncovered relation between excited state dynamics and the molecular packing conditions in this work has direct implications for further optimizing all‐polymer solar cells based on PSMAs. As summarized in Table S3 (Supporting Information), the PCE of PBDB‐T: PJ1 is 14.4%, which is slightly higher than that (14.0%) of PBDB‐T: Y5.^[^ ^31^, ^53^, ^54^ ^]^ The PCE increasement of PBDB‐T: PJ1 are mainly because of the higher *V*_oc_, which is consistent with the suppressed non‐radiative triplet loss during charge recombination. Nevertheless, the short‐circuit current density (*J*_sc_) of PBDB‐T: PJ1 lags behind PBDB‐T: Y5, which may be because of the additional loss from LEs in PJ1. To achieve higher PCEs in PSMA based devices, charge generation and recombination should be simultaneously optimized like state‐of‐the‐art OPV devices based on SMAs of Y6 and its derivatives.^[^ ^55^, ^56^ ^]^ In PSMA films, the conversion from LE to i‐DE is largely suppressed in comparison with SMA films. The generation of i‐DE is found related to the coherent length and packing probability of *π*–*π* stacking in PSMA.^[^ ^28^, ^57^ ^]^ Therefore, two possible methods such as introducing strong electron withdrawing group to increase the push‐pulling effect and modifying the side chains and linking units to increase the interchain *π*–*π* stacking may help to expand the fraction of i‐DE states after photon excitation. Thermal annealing is often used to fine tuning the micromorphology of phase separation and *π*–*π* stacking.^[^ ^58^ ^]^ However, we find in blends with PSMAs, the excited dynamics of the blend based on PSMA are annealing insensitive (Figure S12, Supporting Information), which further increase the difficulty for optimizing the device performance based on PSMA by merely thermal annealing. To solve the problem, other methods such as adding additives, sequential deposition, and variation of processing solvent may be used together with thermal annealing to fine control the molecular packing of PSMAs.^[^ ^59^, ^60^, ^61^ ^]^ Nevertheless, more work should be done to better understand the mechanism between the micromorphology of molecular packing and the excited‐state dynamics in polymer blends with PSMAs.

## 3 Conclusion

In summary, we have observed that SMA polymerization decelerates the conversion from LEs to i‐DEs in films of PMSAs. In OPV blends with PSMAs, the i‐DE mediated charge separation is faster, and the nonradiative charge recombination of triplet formation is largely suppressed, but the reduced LE to i‐DE conversion leads to more radiative loss from LE. The decelerated LE to i‐DE conversion and suppressed charge recombination can be explained by decreased *π*–*π* stacking and reduced D: A interfaces in PSMA based blends, as supported by GIWAXS and MD simulation results. Our findings highlight the effect of changed molecular arrangement after SMA polymerization on the excited state dynamics in OPV blends with PSMAs, indicating that modifying the *π*–*π* interaction between interchain subunits in PSMAs is a promising route to optimize the charge generation and recombination dynamics for high performance all‐PSCs.

## Conflict of Interest

The authors declare no conflict of interest.

## Supporting information

Supporting Information

## Data Availability

The data that support the findings of this study are available from the corresponding author upon reasonable request.

## References

[1] Y. Xu , J. Yuan , S. Zhou , M. Seifrid , L. Ying , B. Li , F. Huang , G. C. Bazan , W. Ma , Adv. Funct. Mater. 2019, 29, 1806747.

[2] Y. Zhang , Y. Xu , M. J. Ford , F. Li , J. Sun , X. Ling , Y. Wang , J. Gu , J. Yuan , W. Ma , Adv. Energy Mater. 2018, 8, 1800029.

[3] G. Wang , F. S. Melkonyan , A. Facchetti , T. J. Marks , Angew. Chem., Int. Ed. 2019, 58, 4129. 10.1002/anie.201808976 30395372

[4] Q. Fan , Z. Xiao , E. Wang , L. Ding , Sci. Bull. 2021, 66, 1950. 10.1016/j.scib.2021.07.002 36654163

[5] T. Kim , J.‐H. Kim , T. E. Kang , C. Lee , H. Kang , M. Shin , C. Wang , B. Ma , U. Jeong , T.‐S. Kim , B. J. Kim , Nat. Commun. 2015, 6, 8547. 26449658 10.1038/ncomms9547 PMC4633811

[6] Z. Genene , W. Mammo , E. Wang , M. R. Andersson , Adv. Mater. 2019, 31, 1807275. 10.1002/adma.201807275 30790384

[7] C. Lee , S. Lee , G.‐U. Kim , W. Lee , B. J. Kim , Chem. Rev. 2019, 119, 8028. 31181904 10.1021/acs.chemrev.9b00044

[8] Z.‐G. Zhang , Y. Li , Angew. Chem., Int. Ed. 2021, 60, 4422. 10.1002/anie.202009666 32815309

[9] Z.‐G. Zhang , Y. Yang , J. Yao , L. Xue , S. Chen , X. Li , W. Morrison , C. Yang , Y. Li , Angew. Chem., Int. Ed. 2017, 56, 13503. 10.1002/anie.201707678 28856814

[10] R. Sun , T. Wang , Q. Fan , M. Wu , X. Yang , X. Wu , Y. Yu , X. Xia , F. Cui , J. Wan , X. Lu , X. Hao , A. K. Y. Jen , E. Spiecker , J. Min , Joule 2023, 7, 221.

[11] R. Ma , J. Yu , T. Liu , G. Zhang , Y. Xiao , Z. Luo , G. Chai , Y. Chen , Q. Fan , W. Su , G. Li , E. Wang , X. Lu , F. Gao , B. Tang , H. Yan , Aggregate 2022, 3, e58.

[12] Q. Ma , Z. Jia , L. Meng , H. Yang , J. Zhang , W. Lai , J. Guo , X. Jiang , C. Cui , Y. Li , Adv. Funct. Mater. 2023, 33, 2210733.

[13] X. Yang , R. Sun , Y. Wang , M. Chen , X. Xia , X. Lu , G. Lu , J. Min , Adv. Mater. 2022, 35, 2209350. 10.1002/adma.202209350 36413076

[14] J. Ji , L. Zhu , X. Xiong , F. Liu , Z. Liang , Adv. Sci. 2022, 9, 2200864. 10.1002/advs.202200864 PMC9313542 35595683

[15] D. Zhou , C. Liao , S. Peng , X. Xu , Y. Guo , J. Xia , H. Meng , L. Yu , R. Li , Q. Peng , Adv. Sci. 2022, 9, 2202022. 10.1002/advs.202202022 PMC9376845 35748169

[16] H. Yu , M. Pan , R. Sun , I. Agunawela , J. Zhang , Y. Li , Z. Qi , H. Han , X. Zou , W. Zhou , S. Chen , J. Y. L. Lai , S. Luo , Z. Luo , D. Zhao , X. Lu , H. Ade , F. Huang , J. Min , H. Yan , Angew. Chem., Int. Ed. 2021, 60, 10137. 10.1002/anie.202016284 33501698

[17] Y. Cui , Y. Xu , H. Yao , P. Bi , L. Hong , J. Zhang , Y. Zu , T. Zhang , J. Qin , J. Ren , Z. Chen , C. He , X. Hao , Z. Wei , J. Hou , Adv. Mater. 2021, 33, 2102420.

[18] R. Sun , Y. Wu , X. Yang , Y. Gao , Z. Chen , K. Li , J. Qiao , T. Wang , J. Guo , C. Liu , X. Hao , H. Zhu , J. Min , Adv. Mater. 2022, 34, 2110147. 10.1002/adma.202110147 35438225

[19] Y. Wei , Z. Chen , G. Lu , N. Yu , C. Li , J. Gao , X. Gu , X. Hao , G. Lu , Z. Tang , J. Zhang , Z. Wei , X. Zhang , H. Huang , Adv. Mater. 2022, 34, 2204718. 10.1002/adma.202204718 35747988

[20] L. Zhu , M. Zhang , J. Xu , C. Li , J. Yan , G. Zhou , W. Zhong , T. Hao , J. Song , X. Xue , Z. Zhou , R. Zeng , H. Zhu , C.‐C. Chen , R. C. I. MacKenzie , Y. Zou , J. Nelson , Y. Zhang , Y. Sun , F. Liu , Nat. Mater. 2022, 21, 656. 35513501 10.1038/s41563-022-01244-y

[21] J. Jin , Q. Wang , K. Ma , W. Shen , L. A. Belfiore , X. Bao , J. Tang , Adv. Funct. Mater. 2023, 33, 2213324.

[22] S. R. Cowan , N. Banerji , W. L. Leong , A. J. Heeger , Adv. Funct. Mater. 2012, 22, 1116.

[23] T. M. Clarke , J. R. Durrant , Chem. Rev. 2010, 110, 6736. 20063869 10.1021/cr900271s

[24] S. M. Ryno , M. K. Ravva , X. Chen , H. Li , J.‐L. Brédas , Adv. Energy Mater. 2017, 7, 1601370.

[25] X.‐K. Chen , M. K. Ravva , H. Li , S. M. Ryno , J.‐L. Brédas , Adv. Energy Mater. 2016, 6, 1601325.

[26] A. Karki , J. Vollbrecht , A. J. Gillett , S. S. Xiao , Y. Yang , Z. Peng , N. Schopp , A. L. Dixon , S. Yoon , M. Schrock , H. Ade , G. N. M. Reddy , R. H. Friend , T.‐Q. Nguyen , Energy Environ. Sci. 2020, 13, 3679.

[27] H. Kang , W. Lee , J. Oh , T. Kim , C. Lee , B. J. Kim , Acc. Chem. Res. 2016, 49, 2424. 27753477 10.1021/acs.accounts.6b00347

[28] R. Wang , C. Zhang , Q. Li , Z. Zhang , X. Wang , M. Xiao , J. Am. Chem. Soc. 2020, 142, 12751. 32602706 10.1021/jacs.0c04890

[29] C. He , Y. Pan , Y. Ouyang , Q. Shen , Y. Gao , K. Yan , J. Fang , Y. Chen , C.‐Q. Ma , J. Min , C. Zhang , L. Zuo , H. Chen , Energy Environ. Sci. 2022, 15, 2537.

[30] J. Hou , O. Inganäs , R. H. Friend , F. Gao , Nat. Mater. 2018, 17, 119. 29358765 10.1038/nmat5063

[31] T. Jia , J. Zhang , W. Zhong , Y. Liang , K. Zhang , S. Dong , L. Ying , F. Liu , X. Wang , F. Huang , Y. Cao , Nano Energy 2020, 72, 104718.

[32] M. Kataria , H. D. Chau , N. Y. Kwon , S. H. Park , M. J. Cho , D. H. Choi , ACS Energy Lett. 2022, 7, 3835.

[33] G. Li , X. Zhang , L. O. Jones , J. M. Alzola , S. Mukherjee , L.‐w. Feng , W. Zhu , C. L. Stern , W. Huang , J. Yu , V. K. Sangwan , D. M. DeLongchamp , K. L. Kohlstedt , M. R. Wasielewski , M. C. Hersam , G. C. Schatz , A. Facchetti , T. J. Marks , J. Am. Chem. Soc. 2021, 143, 6123. 33848146 10.1021/jacs.1c00211

[34] E. A. Chandross , J. Ferguson , J. Chem. Phys. 1967, 47, 2557.

[35] N. J. Hestand , F. C. Spano , Chem. Rev. 2018, 118, 7069. 29664617 10.1021/acs.chemrev.7b00581

[36] G. Han , Y. Yi , J. Phys. Chem. Lett. 2019, 10, 2911. 31088080 10.1021/acs.jpclett.9b00928

[37] R. Steyrleuthner , M. Schubert , I. Howard , B. Klaumünzer , K. Schilling , Z. Chen , P. Saalfrank , F. Laquai , A. Facchetti , D. Neher , J. Am. Chem. Soc. 2012, 134, 18303. 22957688 10.1021/ja306844f

[38] Z. Zheng , H. Yao , L. Ye , Y. Xu , S. Zhang , J. Hou , Mater. Today 2020, 35, 115.

[39] S. J. Yoon , K. S. Choi , L. Zhong , S. Jeong , Y. Cho , S. Jung , S. E. Yoon , J. H. Kim , C. Yang , Small 2023, 2300507. 10.1002/smll.202300507 37010009

[40] S. Jeong , J. Park , Y. Ji , Y. Cho , B. Lee , M. Jeong , S. Jung , S. Yang , Y. Zhang , S.‐J. Yoon , C. Yang , J. Mater. Chem. A 2023, 11, 4703.

[41] C. C. S. Chan , C. Ma , X. Zou , Z. Xing , G. Zhang , H.‐L. Yip , R. A. Taylor , Y. He , K. S. Wong , P. C. Y. Chow , Adv. Funct. Mater. 2021, 31, 2107157.

[42] A. J. Gillett , A. Privitera , R. Dilmurat , A. Karki , D. Qian , A. Pershin , G. Londi , W. K. Myers , J. Lee , J. Yuan , S.‐J. Ko , M. K. Riede , F. Gao , G. C. Bazan , A. Rao , T.‐Q. Nguyen , D. Beljonne , R. H. Friend , Nature 2021, 597, 666. 34588666 10.1038/s41586-021-03840-5

[43] R. Wang , J. Xu , L. Fu , C. Zhang , Q. Li , J. Yao , X. Li , C. Sun , Z.‐G. Zhang , X. Wang , Y. Li , J. Ma , M. Xiao , J. Am. Chem. Soc. 2021, 143, 4359. 33719415 10.1021/jacs.0c13352

[44] A. Rao , P. C. Y. Chow , S. Gélinas , C. W. Schlenker , C.‐Z. Li , H.‐L. Yip , A. K. Y. Jen , D. S. Ginger , R. H. Friend , Nature 2013, 500, 435. 23925118 10.1038/nature12339

[45] G. Lakhwani , A. Rao , R. H. Friend , Annu. Rev. Phys. Chem. 2014, 65, 557. 24423376 10.1146/annurev-physchem-040513-103615

[46] J. Benduhn , K. Tvingstedt , F. Piersimoni , S. Ullbrich , Y. Fan , M. Tropiano , K. A. McGarry , O. Zeika , M. K. Riede , C. J. Douglas , S. Barlow , S. R. Marder , D. Neher , D. Spoltore , K. Vandewal , Nat. Energy 2017, 2, 17053.

[47] Z. Liu , Z. Liu , R. Wang , Z.‐G. Zhang , J. Wang , C. Zhang , J. Phys. Chem. Lett. 2022, 13, 10305. 36305820 10.1021/acs.jpclett.2c03020

[48] F. Etzold , I. A. Howard , N. Forler , D. M. Cho , M. Meister , H. Mangold , J. Shu , M. R. Hansen , K. Müllen , F. Laquai , J. Am. Chem. Soc. 2012, 134, 10569. 22612417 10.1021/ja303154g

[49] W. Zhong , M. Zhang , L. Zhu , Y. Zhang , F. Liu , Trends Chem 2022, 4, 699.

[50] G. Han , Y. Guo , X. Ma , Y. Yi , Sol. RRL 2018, 2, 1800190.

[51] G. Zhang , X.‐K. Chen , J. Xiao , P. C. Y. Chow , M. Ren , G. Kupgan , X. Jiao , C. C. S. Chan , X. Du , R. Xia , Z. Chen , J. Yuan , Y. Zhang , S. Zhang , Y. Liu , Y. Zou , H. Yan , K. S. Wong , V. Coropceanu , N. Li , C. J. Brabec , J.‐L. Bredas , H.‐L. Yip , Y. Cao , Nat. Commun. 2020, 11, 3943. 32770068 10.1038/s41467-020-17867-1 PMC7414148

[52] K. Jiang , J. Zhang , C. Zhong , F. R. Lin , F. Qi , Q. Li , Z. Peng , W. Kaminsky , S.‐H. Jang , J. Yu , X. Deng , H. Hu , D. Shen , F. Gao , H. Ade , M. Xiao , C. Zhang , A. K. Y. Jen , Nat. Energy 2022, 7, 1076.

[53] J. Yuan , Y. Zhang , L. Zhou , C. Zhang , T.‐K. Lau , G. Zhang , X. Lu , H.‐L. Yip , S. K. So , S. Beaupré , M. Mainville , P. A. Johnson , M. Leclerc , H. Chen , H. Peng , Y. Li , Y. Zou , Adv. Mater. 2019, 31, 1807577. 10.1002/adma.201807577 30883937

[54] Q. Fan , Q. An , Y. Lin , Y. Xia , Q. Li , M. Zhang , W. Su , W. Peng , C. Zhang , F. Liu , L. Hou , W. Zhu , D. Yu , M. Xiao , E. Moons , F. Zhang , T. D. Anthopoulos , O. Inganäs , E. Wang , Energy Environ. Sci. 2020, 13, 5017.

[55] J. Yuan , Y. Zhang , L. Zhou , G. Zhang , H.‐L. Yip , T.‐K. Lau , X. Lu , C. Zhu , H. Peng , P. A. Johnson , M. Leclerc , Y. Cao , J. Ulanski , Y. Li , Y. Zou , Joule 2019, 3, 1140.

[56] H. Yao , J. Hou , Angew. Chem., Int. Ed. 2022, 61, e202209021. 10.1002/anie.202209021 35853834

[57] Z. Guo , D. Lee , R. D. Schaller , X. Zuo , B. Lee , T. Luo , H. Gao , L. Huang , J. Am. Chem. Soc. 2014, 136, 10024. 24956140 10.1021/ja503465s

[58] F. Zhao , C. Wang , X. Zhan , Adv. Energy Mater. 2018, 8, 1703147.

[59] Q. Wu , W. Wang , Y. Wu , Z. Chen , J. Guo , R. Sun , J. Guo , Y. Yang , J. Min , Adv. Funct. Mater. 2021, 31, 2010411.

[60] W. Wang , Q. Wu , R. Sun , J. Guo , Y. Wu , M. Shi , W. Yang , H. Li , J. Min , Joule 2020, 4, 1070.

[61] Q. Wu , W. Wang , T. Wang , R. Sun , J. Guo , Y. Wu , X. Jiao , C. J. Brabec , Y. Li , J. Min , Sci. China Chem. 2020, 63, 1449.

