# Supplementary material for: Excited‐State Dynamics in All‐Polymer Blends with Polymerized Small‐Molecule Acceptors
Source: Adv Sci (Weinh). 2023 Jun 4;10(23):2301931. doi: 10.1002/advs.202301931 (PMC10427414; doi:10.1002/advs.202301931)
Supplement: Supplementary file 1 — Supporting Information [file ADVS-10-2301931-s001.pdf]

ADVANCED SCIENCE
Open Access

Supporting Information

for *Adv. Sci.*, DOI 10.1002/advs.202301931

Excited-State Dynamics in All-Polymer Blends with Polymerized Small-Molecule Acceptors

Ziran Liu, Qian Li, Lulu Fu, Jide Wang, Jing Ma, Chunfeng Zhang* and Rui Wang**

Excited-State Dynamics in All-polymer Blends with Polymerized Small-Molecule Acceptors

Ziran Liu, [#] *Qian Li,* [#] *Lulu Fu,*[#] *Jide Wang, Jing Ma,* [*] *Chunfeng Zhang,*[*] and *Rui Wang*[*].

Z. Liu, J. Wang
Key Laboratory of Oil and Gas Fine Chemicals, Ministry of Education & Xinjiang Uygur Autonomous Region, School of Chemical Engineering and Technology, Xinjiang University, Urumqi 830046, China.
Z. Liu, Q. Li, Prof. C. Zhang
National Laboratory of Solid State Microstructures, School of Physics, and Collaborative Innovation Center for Advanced Microstructures, Nanjing University, Nanjing 210093, China.
E-mail: cfzhang@nju.edu.cn
L. Fu
School of Materials Science and Engineering, Qilu University of Technology (Shandong Academy of Sciences), Jinan 250000, China.
Prof. J. Ma
Institute of Theoretical and Computational Chemistry, Key Laboratory of Mesoscopic Chemistry of MOE, School of Chemistry and Chemical Engineering, Nanjing University, Nanjing 210093, China.
E-mail: majing@nju.edu.cn
Prof. C. Zhang
Institute of Materials Engineering, Nanjing University, Nantong, Jiangsu, China.
Prof. R. Wang
College of Physics, Nanjing University of Aeronautics and Astronautics, and Key Laboratory of Aerospace Information Materials and Physics (NUAA), MIIT, Nanjing 211106, China.
E-mail: rui_wang@nuaa.edu.cn

[#] These authors contributed equally to this work.

Keywords: Excited-state dynamics, polymerized small molecular acceptor, π-π stacking, transient absorption, charge generation and recombination

Abstract: Polymerizing small-molecular acceptors (SMAs) is a promising route to construct high performance polymer acceptors of all-polymer solar cells (all-PSCs). After SMA polymerization, the microstructure of molecular packing is largely modified, which is essential in regulating the excited-state dynamics during the photon-to-current conversion. Nevertheless, the relationship between the molecular packing and excited-state dynamics in polymerized SMAs (PSMAs) remains poorly understood. Herein, we investigate the excited-state dynamics and molecular packing in the corresponding PSMA and SMA utilizing a combination of experimental and theoretical methods. We find that the charge separation from intra-moiety delocalized states (i-DEs) is much faster in blends with PSMAs, but the loosed π-π molecular packing suppresses the excitation conversion from the local excitation (LE) to the i-DE, leading to additional radiative losses from LEs. Moreover, the increased aggregations of PSMA in the blends decrease donor: acceptor interfaces, which reduces triplet losses from the bimolecular charge recombination. These findings suggest that excited-state dynamics may be manipulated by the molecular packing in blends with PSMAs to further optimize the performance of all-PSCs.

Sample preparation

The materials of donors PBDB-T and acceptor Y5 were purchased from Solarmer Materials Inc. PJ1 was synthesized by the methods reported in the literature.[1] The film samples for transient absorption (TA) measurements were prepared using the approach of spin coating. The blend samples were made with 1:1.5 weight ratio of donor/acceptor and with thermal annealing at 100 °C for 10 min for the PJ1-based blends and at 110 °C for 10 min for the Y5-based blends. The film thicknesses were ~ 100 nm. The solution samples were prepared by desolving PJ1 or Y5 into toluene in a 1 mm cuvette. The concentration was ~ 0.1 mmol L^{-1}.

Optical characterizations

The fs-TA measurements were conducted using a Yb:KGW laser (Pharos, Light Conversion). The wavelength of the fundamental output was at ~1,030 nm. We used a home-built noncollinear optical parametric amplifier to generate the pump pulses around 800 nm. The probe beam was supercontinuum by focusing a small fraction of the fundamental 1030 beam to a 5 mm sapphire plate for visible detection or a 6 mm yttrium aluminium garnet (YAG) plate

for infrared detection. A short pass filter (10SWF-1000-B, Newport) or a long pass filter (FELH1100, Thorlabs) was employed in the supercontinuum to block the fundamental beam for visible detection (550-950 nm) or infrared detection (1,100-1,600 nm), respectively. The pump and visible probe pulses were compressed by chirp mirrors (DCM 9, Laser Quantum) equipped with wedge pairs, and the near infrared probe was compressed by a deformation mirror (MMDM, Flexible Optical B. V). The overall time resolution of the TA system was better than 30 fs. The supercontinuum light was split into two beams for balanced detection. The probe and reference beams were then routed to either a double-line Si camera (S14417, Hamamatsu) for visible detection or a double-line InGaAs camera (G11608, Hamamatsu) for infrared detection. The cameras were mounted on a monochromator (Acton 2358, Princeton Instrument). Pulse-to-pulse spectral analysis was conducted at 50 kHz for visible detection and 16 kHz for infrared detection using a homemade field-programmable gate array (FPGA) control board. The signal-to-noise ratio ($\Delta T/T$) was better than 1×10^{-5} after averaging 25,000 pump-on and pump-off shots for each data point. In the ns-TA measurements, the pump laser was replaced by a pulsed laser diode emitted at 670 nm (LDH-P-C-670M, Picoquant). The time delay between the two lasers was synchronized by a digital delay generator (DG645, Stanford Research System). The pump fluence was set at 2 μJ cm^{-2} unless otherwise specified. The samples were kept in a nitrogen atmosphere during the measurements to prevent photo-degradation.

Triplet sensitization measurements

The spectral features of the triplet states in the blends were characterized by the well-established approach of triplet sensitization[2]. We used platinum-octaethyl-porphyrin (PtOEP) as the sensitizer in film samples with optical excitation at 385 nm. The pump photon energy mainly excites the sensitizer, and the triplet state of PtOEP (~ 1.9 eV) is populated by the process of intersystem crossing. The triplets of PtOEP then transfer to the acceptors, and the excited-state absorption (ESA) feature of the T_1 state in the acceptors can be captured by the transient absorption spectra (Figure S6). The sensitized triplet absorption spectra are well correlated to the long-lived states in PBDB-T:Y5 and PBDB-T:PJ1 blends, which indicates the long-lived features can be assigned as triplet excitons in the acceptors.

Computational details

The intermolecular packing morphology of donor and acceptor molecules in the blend

films has been explored by the molecular dynamics (MD) simulations. All-atom MD simulations were carried out by using the package suite of AMBER 16 CUDA version.[3-5] The atom types, intra- and intermolecular interaction parameters were built from the general AMBER force field (GAFF).[6] The initial packing models of PBDB-T/acceptor were constructed by randomly mixing 40 PBDB-T with 8 repeat units (n=8) and acceptor (31 PJ1 with 8 repeat unit, or 343 Y5) in 1:1 weight ratio to model the experiment condition, which were then placed into the periodic box of dimension 350 Å × 350 Å × 350 Å. The blends experienced a transient minimization before MD simulations. To simulate the process of thermal annealing in experiments, the MD simulations were performed at different temperatures, heating from 298 K to 373 K within 100 ps, respectively, then equilibration at 373 K with duration of 2 ns, finally decreasing from 373 K to 298 K within 200 ps for PBDB-T: PJ1. Finally, the final equilibration under NPT (constant atom numbers, pressure of 1 atom, and temperature of 298 K) ensemble lasts for 80 ns, for the purpose of trajectory production and analysis. Different from PBDB-T: PJ1 systems, the thermal annealing temperature is set at 383 K for PBDB-T: Y5 blends.

The center-of-mass radial distribution function (RDF) was calculated between different molecules or molecular fragment of simulated samples from the last 20 ns trajectories. In the analysis of RDF, the side chains of investigated particles are not taken into the account. It has been supposed that the frontier orbitals of acceptor are not distributing over the side chains, which are not expected to be involved into the charge-transport process.[4-5] The calculation of RDF from MD trajectory data was implemented on Visual Molecular Dynamics (VMD)[6].

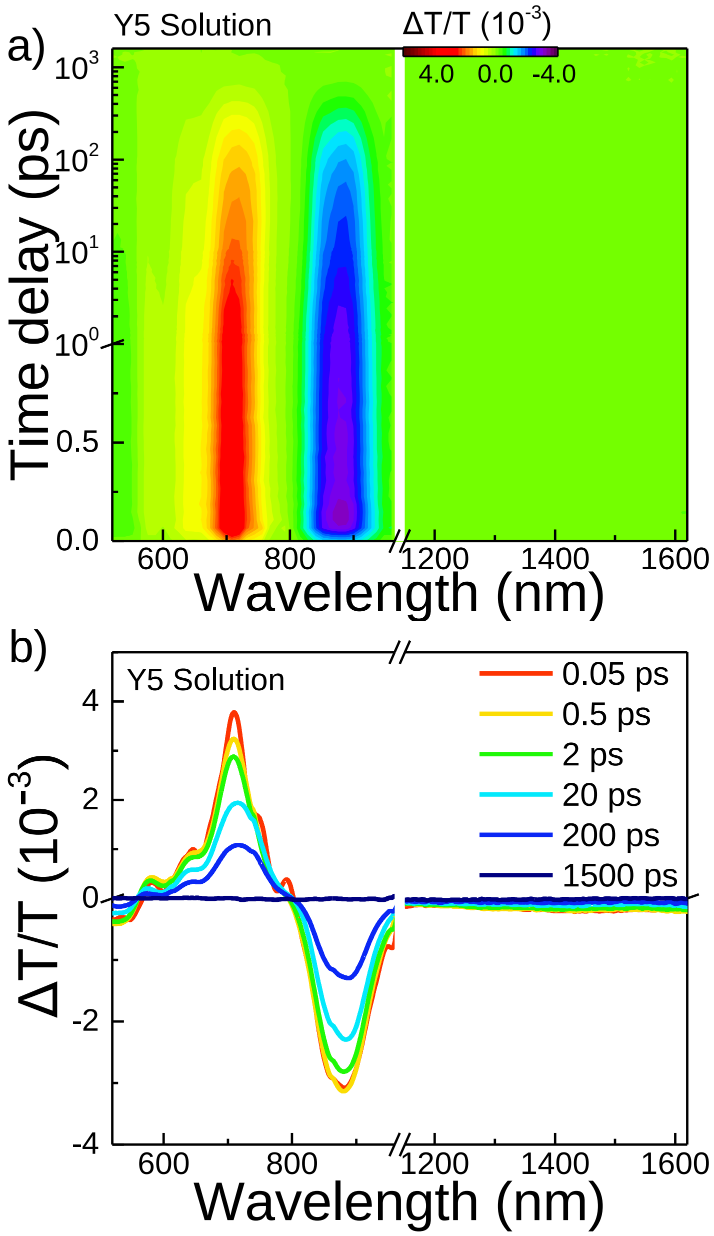

Figure S1. (a) Transient absorption data recorded from the sample of Y5 solution. (b) Transient absorption spectra at different time delays of Y5 solution. The ESA feature is similar to the feature probed at ~900 nm recorded from the Y5 film except for a slight wavelength shift.

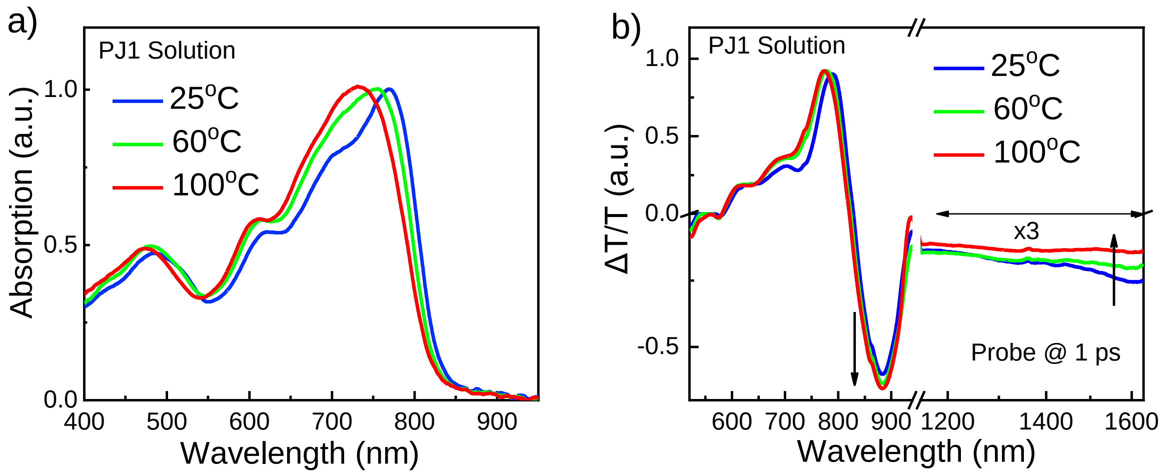

Figure S2. (a) Absorption spectra of the PJ1 solutions in toluene with different temperatures. The bule-shifted absorption peak suggests the π-π packing is reduced with increasing temperatures. (b) The ESA signals around 1580 nm decrease under high temperatures, demonstrating the conversion from LE to i-DE is suppressed with the reduced π-π stacking.

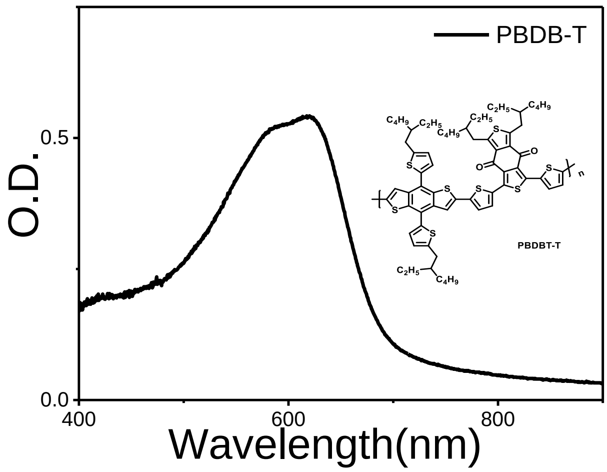

Figure S3. Molecular structures and absorption spectra of a PBDB-T neat film.

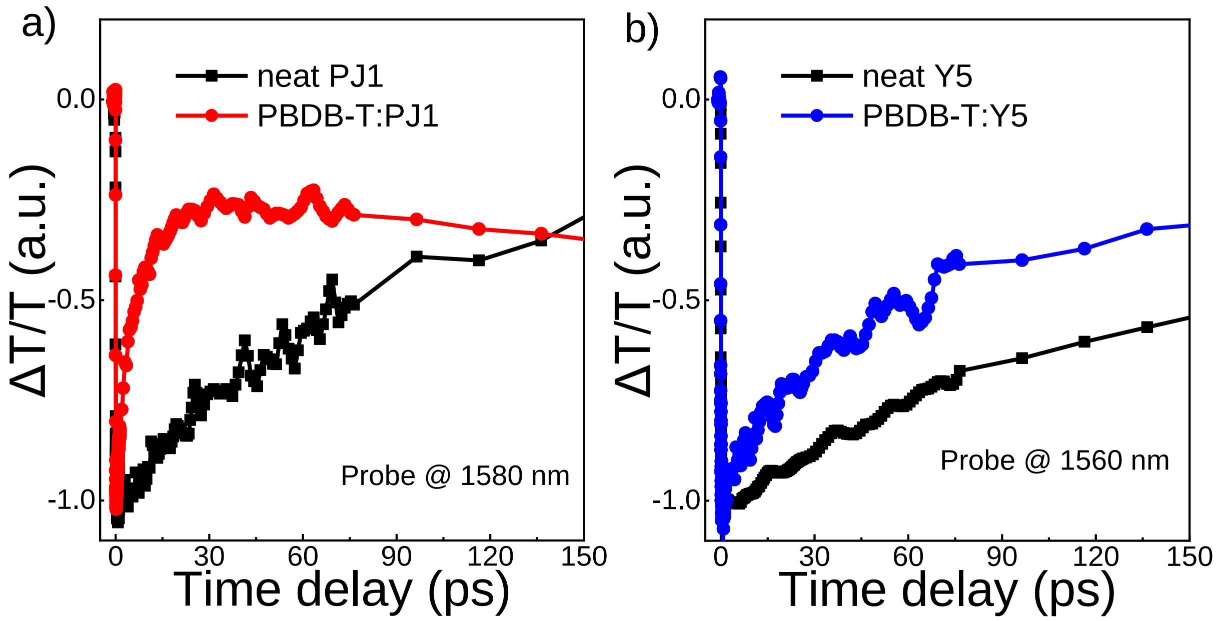

Figure S4. (a-b) Normalized TA traces probed at 1580 nm and 1560 nm recorded from blends and neat films with PJ1 and Y5, respectively. The dynamics of i-DE around 1580 nm and 1560 nm probe decay much faster in the two blends, demonstrating the i-DE mediated hole transfer process.

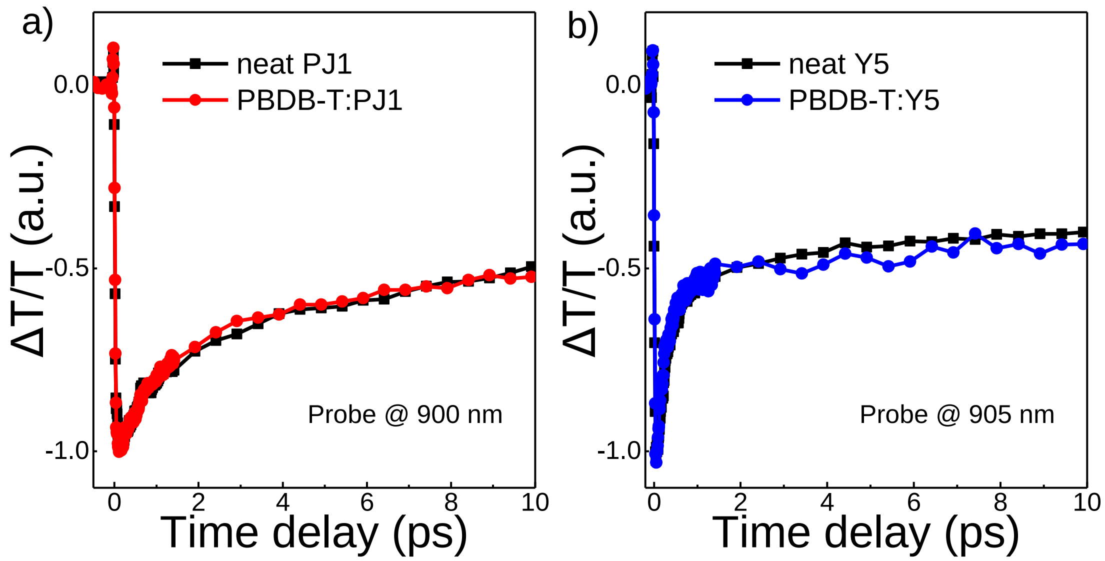

Figure S5. (a-b) Normalized TA traces probed around 900 nm recorded from blends and neat films. The dynamics of LE around 900 nm probe barely changed between neat film and blend films, suggesting LE cannot directly undergo hole transfer.

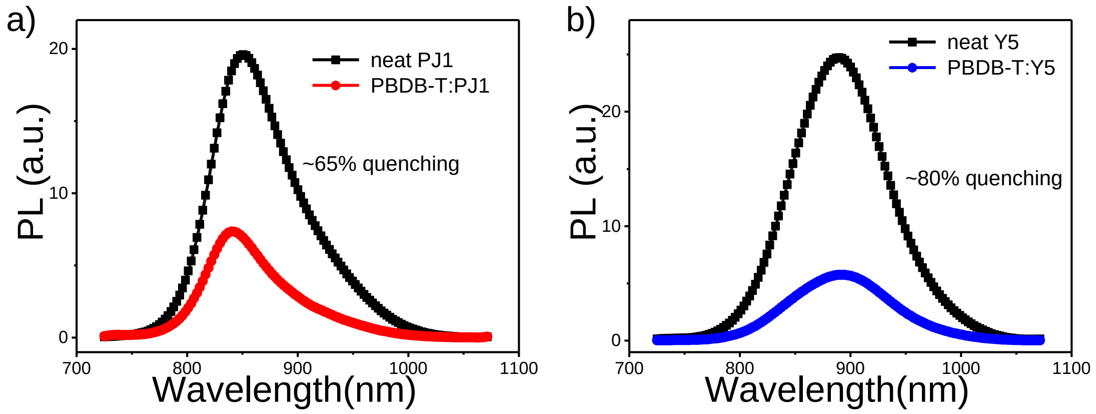

Figure S6. The PL quenching of (a) PBDB-T:PJ1 blend is weaker than (b) PBDB-T:Y5 blend, indicating more radiative recombination from LE in PJ1. The pump wavelength is 670 nm to selectively excite the acceptors.

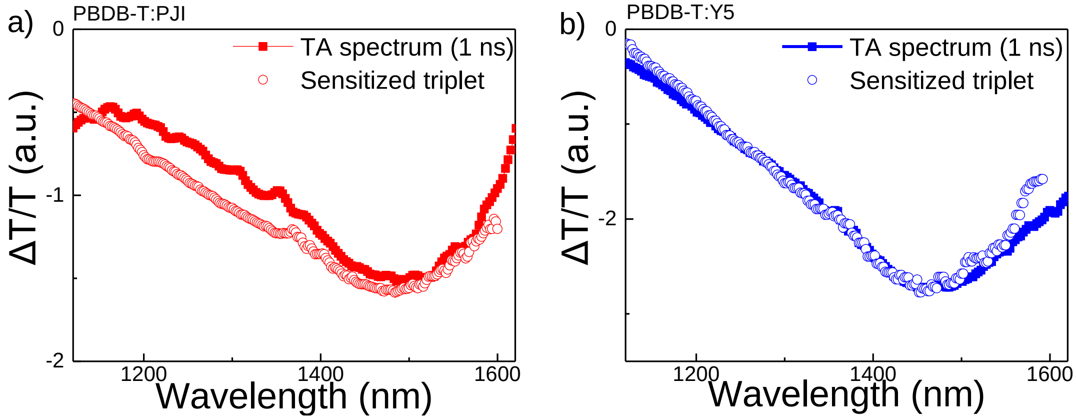

Figure S7. Triplet sensitization. TA spectra of (a) PTOEP/Y5 and (b) PTOEP/PJ1 blends with optical pump at 385 nm compared with the TA spectrum of (a) PBDB-T:Y5 and (b) PBDB-T:PJ1 blends at delay of 1000 ps, respectively. The consistence of the sensitized triplet spectra and the TA spectra at 1000 ps demonstrates the final product in blends is the triplet state.

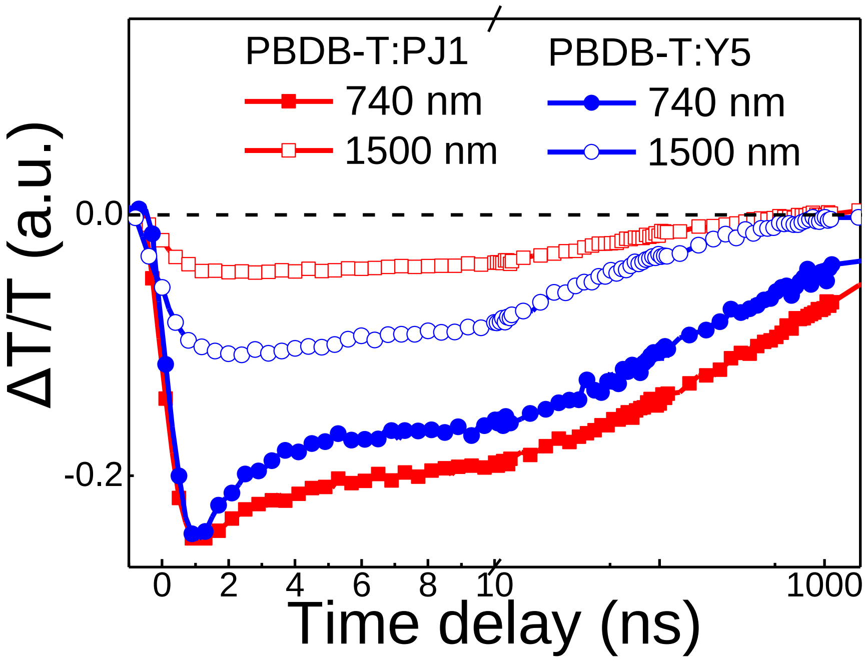

Figure S8. Ns-resolved kinetic curves probed at 740 and 1500 nm recorded from the blend samples with the acceptors of PBDB-T:PJ1 and PBDB-T:Y5, respectively. The signals are normalized at the maximal amplitude of the GSB signal at 800 nm. The data are acquired with pump at 670 nm of 2 μJ/cm^2.

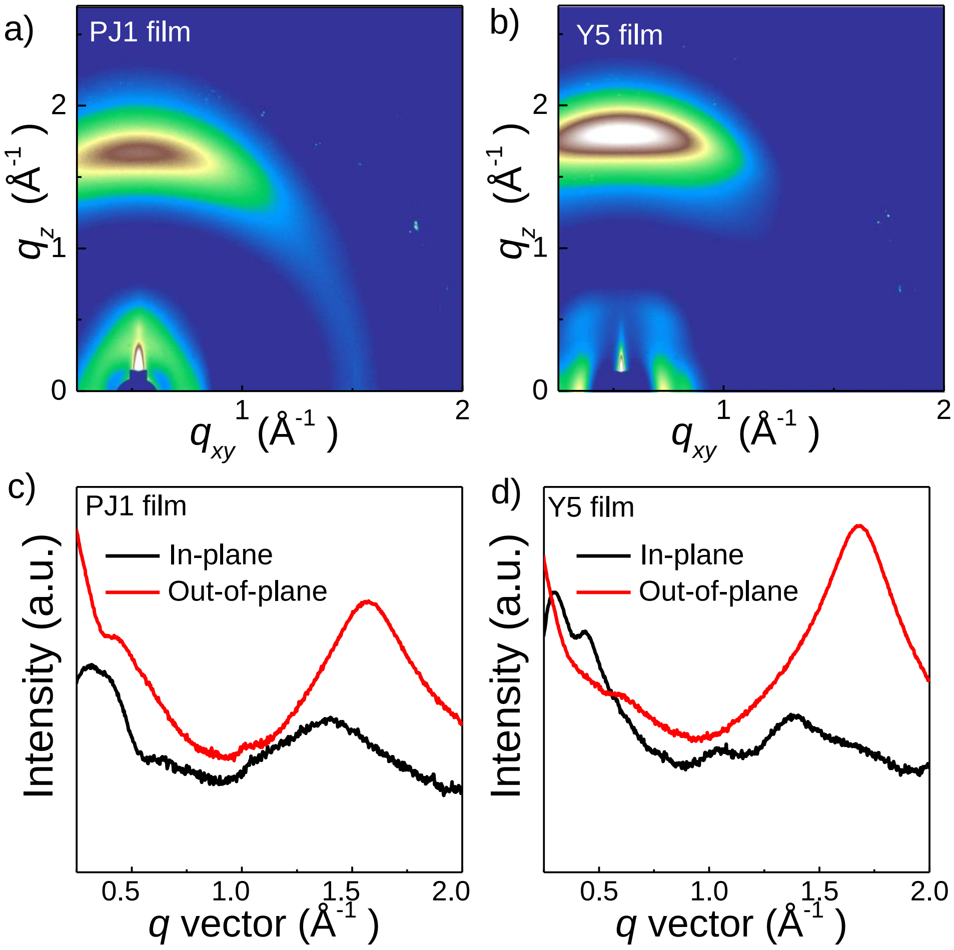

Figure S9. 2D GIWAXS profile of (a) the PJ1 neat film and (b) the Y5 neat film, and (c, d) the corresponding IP and OOP line-cuts from the 2D GIWAXS profiles.

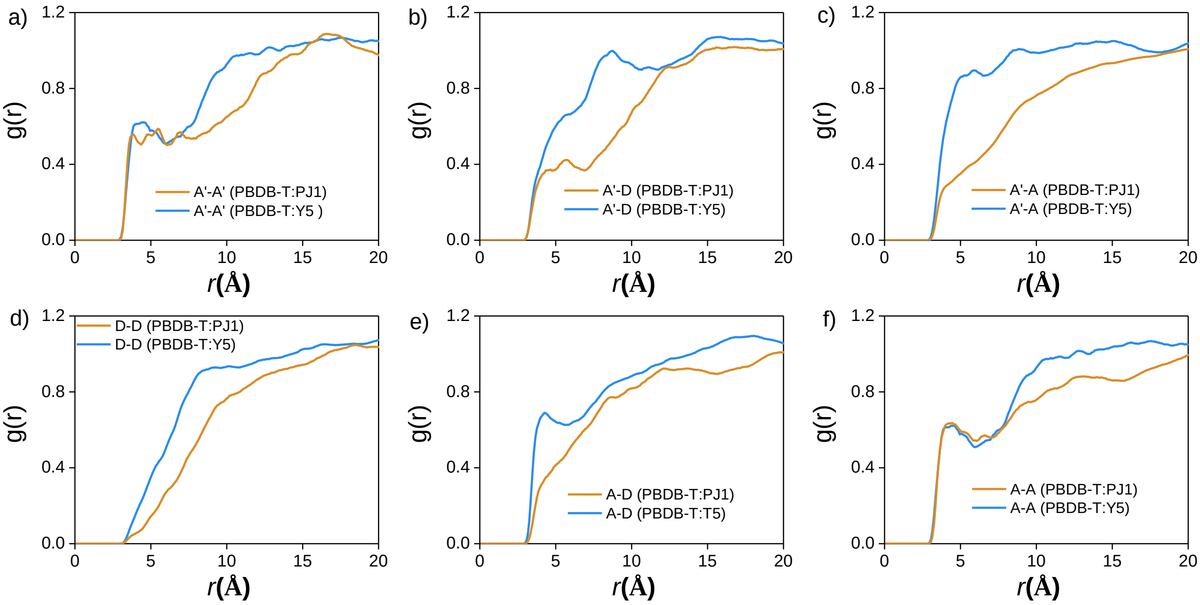

Figure S10. (a–f) Radial distribution function g(r) for the molecular fragments (A, D and A') of PJ1 and Y5 defined in Figure 5a, b extracted from simulated blends. The values RDFs of the PBDB-T:Y5 film are higher than those in PBDB-T:PJ1 blend, indicating that the π - π stacking between the neighboring acceptor molecules in the PBDB-T: Y5 molecular fragments is higher than that in PBDB-T:PJ1.

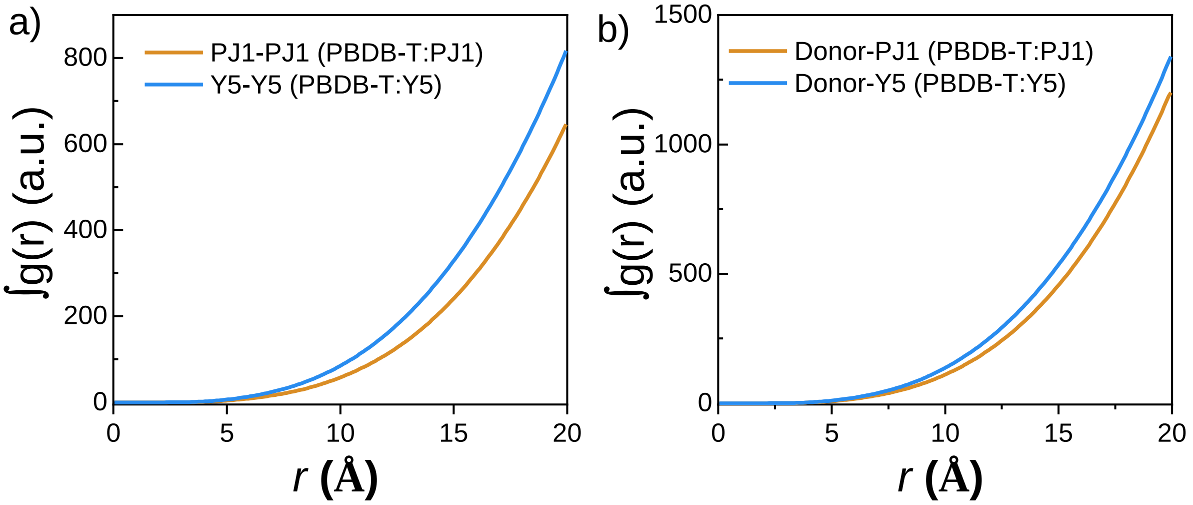

Figure S11. The calculated integration over the distribution of g(r) for (a) PJ1-PJ1 (Y5-Y5) and (b) Donor-PJ1(Y5) in the PBDB-T: acceptor blends.

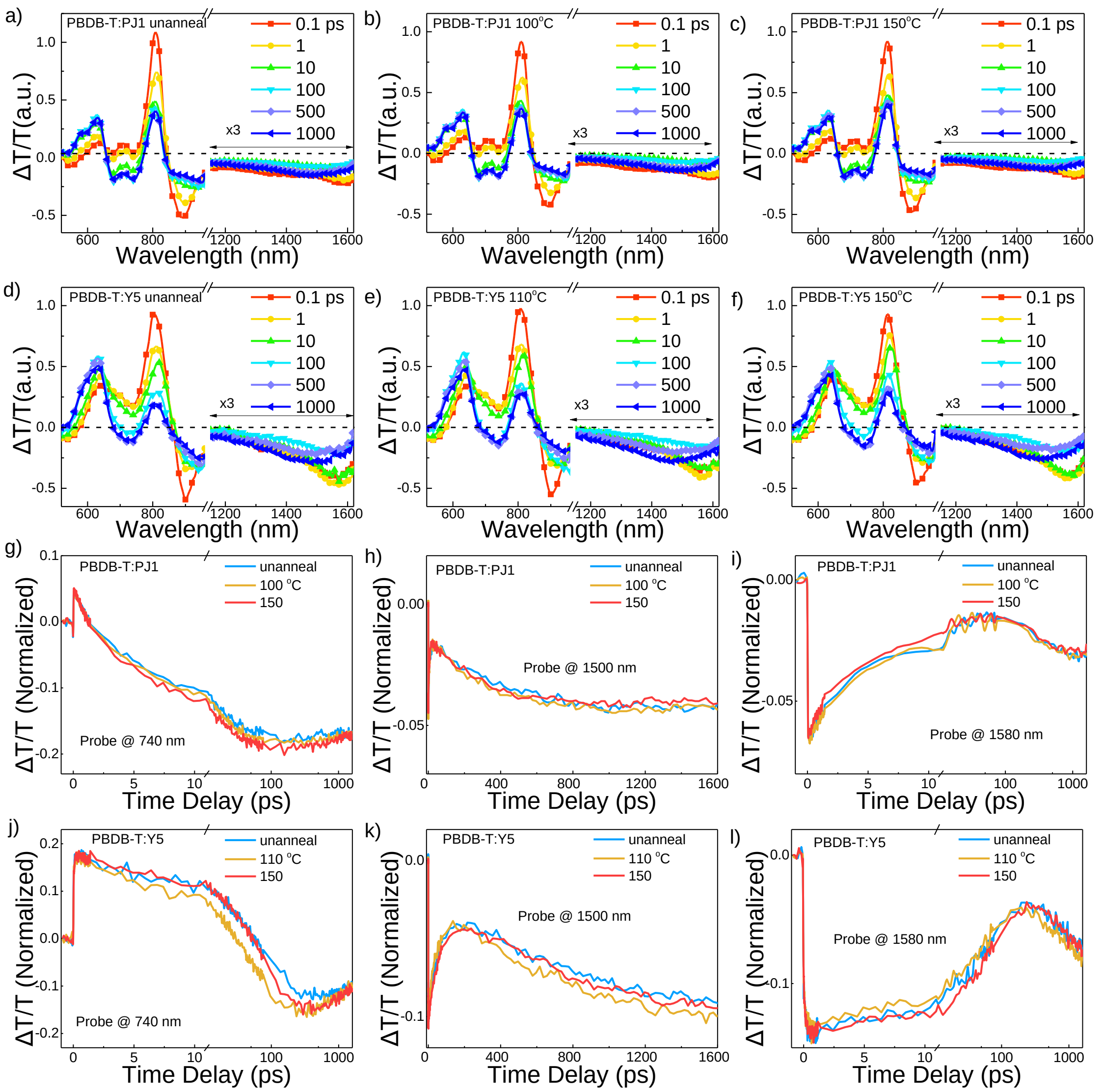

Figure S12. Transient absorption spectra of the film samples of (a-c) PBDB-T:PJ1 and (d-f) PBDB-T:Y5 with different annealing conditions. Dynamics at 740 nm, 1580 nm, and 1500 nm are compared in (g-l) respectively. The TA data are normalized at the initial GSB signals of PJ1and Y5, respectively. The decay dynamics of i-DE at 1580 nm probe keep almost the same in cooperation with the consistent generation of CS states at 740 nm probe in the samples with different annealing conditions. the triplet generation from bimolecular charge recombination at 1500 nm probe are also independent on the sample annealing. These collaborated TA results demonstrate the charge generation and recombination dynamics are morphology insensitive in OPV blends with PSMAs. However, unlike PBDB-T:PJ1, we find the charge generation and recombination in PBDB-T:Y5 are both morphology dependent. Under higher annealing temperatures, the PBDB-T:Y5 blends show decelerated charge generation and suppressed triplet loss.

Table S1. The central peak wavelengths of the absorption and emission spectra of PJ1 and Y5 in solutions and films

Sample	PJ1 Solution	PJ1 Film	Y5 Solution	Y5 Film
Absorption peak	770 nm	799 nm	700 nm	790 nm
Emission peak	820 nm	853 nm	738 nm	888 nm

Table S2. Summary of reflection position, d-spacing, and crystal correlation length (CCL) of the indicated films from GIWAXS measurements

Sample	**In plane**				**Out of plane**			
	q_{xy} (Å^{-1})	$d_{lamellar}$ (Å)	FWHM(Å^{-1})	CCL (Å)	q_z (Å-1)	$d_{\pi-\pi}$ (Å)	FWHM (Å^{-1})	CCL (Å)
Y5	0.275	22.863	0.083	68.547	1.681	3.737	0.2	28.644
PJ1	0.287	21.912	0.119	47.482	1.569	4.005	0.258	21.101
PBDB-T:Y5	0.285	22.065	0.064	88.62	1.695	3.708	0.193	29.657
PBDB-T:PJ1	0.287	21.86	0.139	40.797	1.594	3.941	0.295	19.367

Table S3. Photovoltaic Performance of the PBDB-T:Y5 and PBDB-T:PJ1

blends	V_{OC}	J_{SC}	FF	PCEs
PBDB-T:Y5[7]	0.88	22.8	70.2	14%
PBDB-T:PJ1[1]	0.9	22.3	70	14.4%

References:

[1] T. Jia, J. Zhang, W. Zhong, Y. Liang, K. Zhang, S. Dong, L. Ying, F. Liu, X. Wang, F. Huang, Y. Cao, *Nano Energy* **2020**, *72*, 104718.

[2] R. Wang, J. Xu, L. Fu, C. Zhang, Q. Li, J. Yao, X. Li, C. Sun, Z.-G. Zhang, X. Wang, Y. Li, J. Ma, M. Xiao, *J. Am. Chem. Soc.* **2021**, *143*, 4359-4366.

[3] R. Salomon-Ferrer, D. A. Case, R. C. Walker, *WIREs Comput Mol Sci* **2013**, *3*, 198-210.

[4] A. W. Götz, M. J. Williamson, D. Xu, D. Poole, S. Le Grand, R. C. Walker, *J. Chem. Theory Comput.* **2012**, *8*, 1542-1555.

[5] R. Salomon-Ferrer, A. W. Götz, D. Poole, S. Le Grand, R. C. Walker, *J. Chem. Theory Comput.* **2013**, *9*, 3878-3888.

[6] J. Wang, R. M. Wolf, J. W. Caldwell, P. A. Kollman, D. A. Case, *J Comput Chem.* **2004**, *25*, 1157-1174.

[7] J. Yuan, Y. Zhang, L. Zhou, C. Zhang, T.-K. Lau, G. Zhang, X. Lu, H.-L. Yip, S. K. So, S. Beaupré, M. Mainville, P. A. Johnson, M. Leclerc, H. Chen, H. Peng, Y. Li, Y. Zou, *Adv. Mater.* **2019**, *31*, 1807577.
